# Differences in Stability of Viral and Viral-Cellular Fusion Transcripts in HPV-Induced Cervical Cancers

**DOI:** 10.3390/ijms21010112

**Published:** 2019-12-23

**Authors:** Franziska Ehrig, Norman Häfner, Corina Driesch, Irene Kraus Christiansen, Katrin Beer, Martina Schmitz, Ingo B. Runnebaum, Matthias Dürst

**Affiliations:** 1 Department of Gynecology, Jena University Hospital, Friedrich Schiller University Jena, 07747 Jena, Germany; franziska.ehrig@hotmail.de (F.E.); norman.haefner@med.uni-jena.de (N.H.); corina.driesch@uni-jena.de (C.D.); katrin.beer@med.uni-jena.de (K.B.); ingo.runnebaum@med.uni-jena.de (I.B.R.); 2 Department of Microbiology and Infection Control, Akershus University Hospital, 1478 Lørenskog, Norway; Irene.Kraus.Christiansen@ahus.no; 3 Oncgnostics GmbH, 07747 Jena, Germany; martina.schmitz@oncgnostics.com

**Keywords:** cervical cancer, HPV mRNA stability, viral-cellular fusion transcripts

## Abstract

HPV-DNA integration results in dysregulation of viral oncogene expression. Because viral-cellular fusion transcripts inherently lack the viral AU-rich elements of the 3’UTR, they are considered to be more stable than episome-derived transcripts. The aim of this study is to provide formal proof for this assumption by comparing the stability of viral early transcripts derived from episomal and integrated HPV16 DNA, respectively. Full-length cDNA of three fusion transcripts comprising viral and cellular sequences in sense orientation were amplified and cloned into the adeno-viral-vector pAd/CMV/V5-DEST. The most abundant HPV16 oncogene transcript E6*I_-_E7_-_E1_v_E4-E5 with and without 3’UTR, served as reference and control, respectively. Human primary keratinocytes were transduced using high titer virus stocks. qRT-PCR was performed to determine mRNA stability in relation to GAPDH in the presence of actinomycin-D. In four independent transduction experiments, all three viral-cellular fusion transcripts were significantly more stable compared to the episome-derived reference. Among the three viral-cellular fusion transcripts the most stable transcript was devoid of the instability core motif “AUUUA”. Unexpectedly, there was no significant difference in the stability between the episome-derived transcripts either with or without 3’UTR, indicating that the AU-rich elements of the 3’UTR are not contributing to RNA stability. Instead, the three “AUUUA” motifs located in the untranslated region between the viral *E4* and *E5* genes may be responsible for the instability. This is the first report showing that authentic viral-cellular fusion transcripts are more stable than episome-derived transcripts. The longer half-life of the fusion transcripts may result in increased levels of viral oncoproteins and thereby drive the carcinogenic process.

## 1. Introduction

Virus-DNA integration into the host genome is considered to be one of the key events in HPV-induced cervical carcinogenesis [1,2]. In virus-productive cervical lesions (CIN1) the viral genome persists and is replicated as a stable episome. However, in precancerous cervical lesions (CIN3) and, in particular, in cervical carcinomas, the viral DNA is frequently found integrated into the host genome. The integration frequency in cervical cancers differs among the carcinogenic HPV types and is most common for HPV16 and HPV18 [3,4]. Besides tumors which harbor exclusively integrated HPV genomes, tumors with mixed virus states (episomal and integrated) or a solely episomal state are also described in the literature [5,6]. For virus integration to occur, the circular viral genome needs to be linearized. In most cases, the viral non-coding region (ncr) and the viral oncogenes *E6* and *E7* remain intact whereas the *E1* or *E2* regions are frequently disrupted [6,7,8]. This event either uncouples the *E2* gene from the viral promoter p97 or leads to functional inactivation of the affected viral gene itself. In any case, as a consequence, the E2 protein which acts as a negative regulator for viral oncogene transcription cannot be expressed [9,10]. This phenomenon contributes to a deregulated and constitutive expression of the viral oncogenes *E6* and *E7* leading to the inactivation of cell cycle checkpoints via the degradation of p53 and pRB, respectively [11,12]. As a result, cellular mutations, a prerequisite for the development of cancer, accumulate. Moreover, viral integration adjacent to cellular, tissue-specific enhancers, and subsequent co-amplification may also result in high viral oncogene expression [13,14]. Another constellation by which HPV integration may affect the levels of viral oncogenes transcripts was proposed by Jeon and Lambert [15]. They have mapped instability elements (A+U rich elements, ARE) to the 3’UTR of the most common viral oncogene transcript. In their experiments, the HPV16 early 3’UTR sequence was found to be sufficient to confer instability on a heterologous mRNA derived from the human beta-globin gene. Since the viral 3’UTR is not retained in viral-cellular fusion transcripts it was reasoned that these circumstances may result in enhanced mRNA stability and higher levels of oncoprotein [15].

The aim of our study is to provide formal proof for this assumption by comparing the stability of viral early transcripts derived from episomal and integrated HPV16 DNA. For this purpose, the complete cDNA of authentic viral-cellular fusion transcripts identified in three different HPV16-positive cervical carcinomas were cloned into the expression vector (pAd/CMV/V5-DEST) for the transduction of human primary keratinocytes. The most abundant HPV16 oncogene transcript E6*I_-_E7_-_E1_v_E4-E5 with and without 3’UTR served as reference and control, respectively. Overall, we observed that the viral-cellular fusion transcripts were more stable than episome-derived transcripts.

## 2. Results

### 2.1. Recombinant Adenoviruses for Transduction of cDNAs Derived from Integrated and Episomal HPV16 DNA

Complete cDNAs derived from viral-cellular fusion transcripts of three different tumors were amplified by RT-PCR for cloning into the expression vector (pAd/CMV/V5-DEST). Tumors were chosen in which the viral and cellular sequences of the fusion transcripts were both in sense orientation [16,17]. Moreover, in all fusion transcripts, the viral E1 donor site was spliced to a cellular exon site. As a reference, the most abundant HPV16 oncogene transcript, E6*I_-_E7_-_E1_v_E4-E5 with its own 3’UTR, derived from episomal HPV-DNA was also amplified and cloned. The above cDNA without the 3’UTR served as an additional control. The characteristics of all generated recombinant adenoviruses are shown in Table 1.

**Figure 1:**
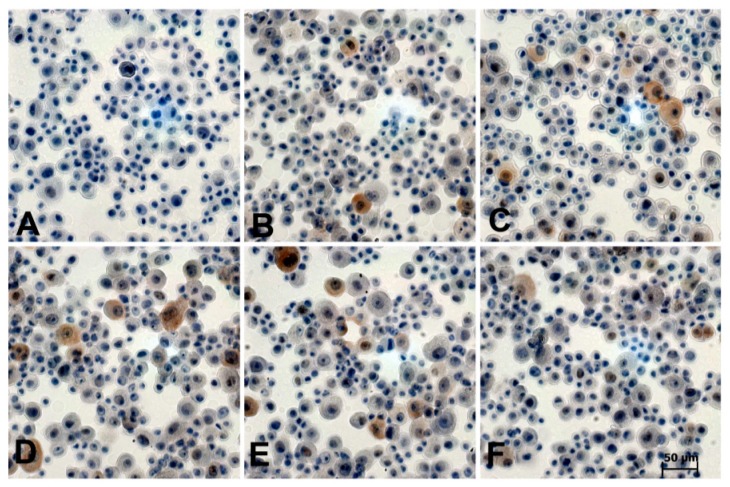
Immunocytochemical staining for HPV16 E7 protein 72 h post-transduction. (**A**) empty vector, (**B**) HPV16epi-plusUTR, (**C**) HPV16epi-minusUTR, (**D**) HPV16-MAP4, (**E**) HPV16-EIF1, (**F**) HPV16-FAM110B. DAB was used as a chromogen. Scale bar for magnification is shown in (**F**).

Of all recombinant adenoviruses, high titer virus stocks (range 10^9^ to 2 × 10^9^) were produced for the transduction of human primary keratinocytes. For transduction, a multiplicity of infection (moi) of 80 was used. The functionality of the recombinant adenoviruses was confirmed by immunocytochemical staining for the HPV-E7 protein 72 h post-transduction (Figure 1). Strong nuclear, as well as combined nuclear and cytoplasmic staining, was observed.

### 2.2. HPV16 Transcript Stability after Transduction of Human Primary Keratinocytes

**Figure 2:**
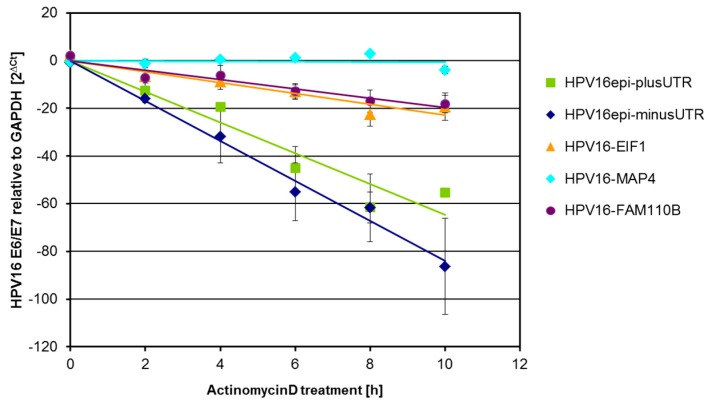
HPV16 transcript stability 72 h post transduction in the presence of actinomycin D. Linear regression of HPV16-E6-E7 transcript levels relative to GAPDH.

Seventy-two hours after transduction, the cells were treated with actinomycin D for up to 10 h to inhibit RNA de-novo synthesis. At intervals of 2 h, total RNA was extracted for the detection of viral oncogene E6-E7 transcripts and GAPDH transcripts. For each transduction experiment, two independent cDNA syntheses were performed, and all PCRs were done in duplicate. Linear regression analyses after normalization to GAPDH are shown in Figure 2 for an exemplary experiment. An increasing or decreasing slope indicates that the mRNA is either more or less stable with respect to GAPDH.

**Figure 3:**
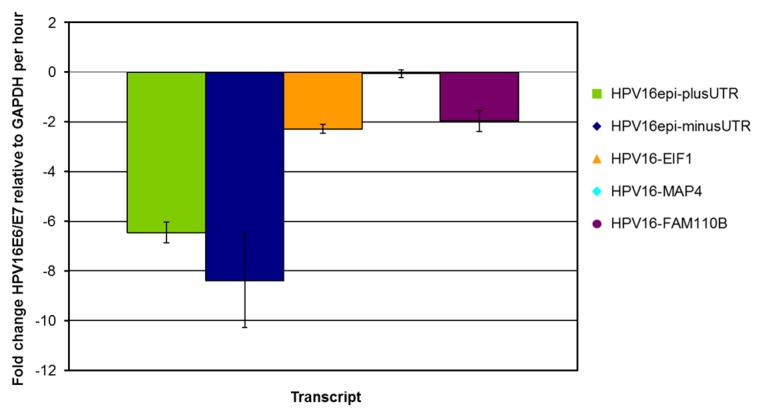
Fold change of HPV16-E6-E7 transcripts relative to GAPDH per hour. Error bars indicate 95% confidence intervals.

The fold change of HPV16-E6-E7 transcripts relative to GAPDH per hour is shown for the same experiment in Figure 3. Clearly, episome derived cDNAs produce less stable transcripts compared to transcripts from integrate-derived viral-cellular fusion cDNAs.

**Figure 4:**
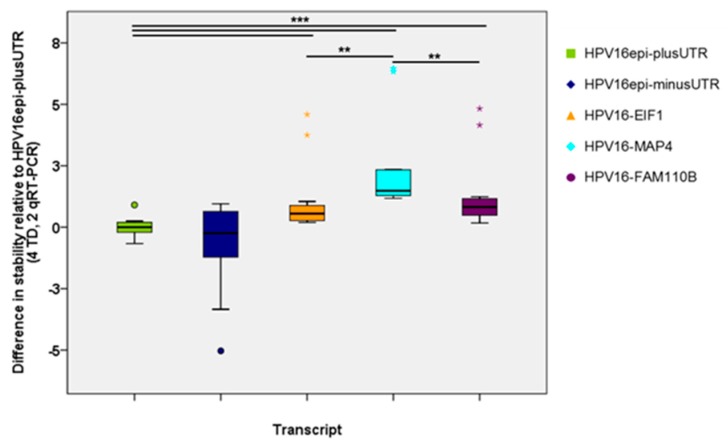
Stability of all transcripts relative to E6*I-E7-E1vE4-E5 with 3’UTR as a reference. The calculation is based on four independent transduction experiments. Statistical significance of the differences was evaluated by Mann–Whitney test (** *p* < 0.01; *** *p* < 0.001).

The stability of all HPV16-E6-E7 transcripts relative to E6*I-E7-E1_V_E4-E5 with 3’UTR (HPV16epi-plus-UTR) as a reference is shown in Figure 4. This analysis included the data of all four independent transduction experiments. E6*I-E7-E1_V_E4-E5 transcripts without 3’UTR (HPV16epi-minus-UTR) exhibit more variable stability than the reference, but this difference is not statistically significant. In contrast, all viral-cellular fusion transcripts are significantly more stable than the reference, whereas HPV16-MAP4 is clearly the most stable one.

### 2.3. Expression at the Protein Level

Although viral-cellular fusion transcripts were shown to be more stable, compared to their solely viral counterparts immunocytochemical staining of transduced cells showed no evident differences in E7 protein expression (Figure 1). Clearly, subtle differences in the expression levels cannot be reliably detected in cytospin preparations. Moreover, the heterogenic pattern of the E7 protein in different subcellular locations poses an additional complication. Besides E7, all transcripts also encode for the spliced isoform of the E6 oncoprotein (E6*I). Unfortunately, immunocytochemical staining for E6*I with two commercially available HPV16-E6 antibodies was not successful.

## 3. Discussion

Dysregulation of viral oncogene expression has been identified as one of the driving factors in cervical carcinogenesis. Normally viral gene expression is tightly regulated by epithelial cell differentiation, hypermethylation, and transcription factors, which may act in concert. This negative regulation may be circumvented by HPV-DNA integration into the host genome. Constitutive expression of the viral oncogenes E6 and E7 in basal and parabasal cells of the infected epithelium causes genetic instability of the host cell—a hallmark of cancer development. Transcriptionally active integrated HPV genomes typically give rise to viral-cellular fusion transcripts whereby viral sequences are often spliced from the E1 splice donor at nt880 to cellular exons [6,17]. Basically, this results in a loss of viral 3’ sequences and may affect RNA stability, as suggested by Jeon and Lambert [15]. However, formal proof for this assumption by comparing the stability of viral early transcripts derived from episomal and integrated HPV16 DNA was thus far lacking. Moreover, the contribution of specific HPV sequences to the instability of solely viral transcripts was not analyzed in detail. Here, we compared the stability of episome-derived transcripts (plus/minus 3’UTR) and viral-cellular fusion transcripts after the transduction of primary keratinocytes and actinomycin D treatment. All fusion transcripts were significantly more stable than solely viral transcripts (Figure 4) providing further support for the hypothesis of increased stability of HPV oncogene transcripts after integration. However, data of the episome-derived transcripts with or without the 3’UTR show similar stability thereby excluding a major contribution of the 3’UTR as suggested by Jeon and Lambert [15]. A possible weakness of the study is that the adenovirus constructs comprise an HCMV promoter which is known to yield high RNA levels. Thus, transcript levels after transduction are likely to be higher compared to endogenous HPV transcripts levels in cervical cancers.

**Figure 5:**
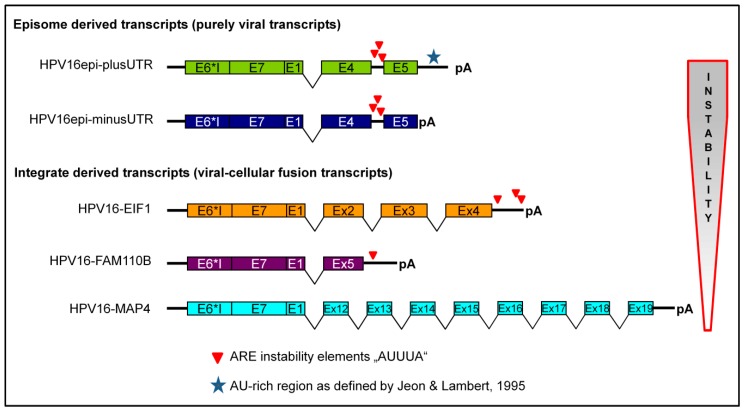
Data summation and interpretation. The observed level of RNA instability (arrow right) correlates with the presence of the AU-rich elements “AUUUA” and not with the AU-rich region denoted with an * in the 3’UTR of purely viral transcripts. Splice sites are symbolized with V and pA refers to polyadenylation.

The stability of RNA is mediated by the recognition of specific sequences either by miRNA or proteins. We have focussed on protein-mediated RNA degradation and analyzed the sequences of both viral and viral-cellular fusion transcripts for the presence of RNA-instability elements. The best-known class of elements influencing RNA stability are AU-rich elements (ARE) [18,19]. They are classified into three major classes: Class 1 and 2 contain dispersed patterns and overlapping repeats of the sequence motif “AUUUA”, respectively, whereas class 3 AREs are less defined and do not contain this motif [20]. Sequence analyses of the transcripts used in this study revealed that the presence of the ARE motif “AUUUA” may contribute to the observed differences in RNA stability (Figure 5). Episome derived transcripts contain three “AUUUA” motifs within a 220 bp sequence located between the E4 and E5 open reading frames. All integrate-derived fusion transcripts are devoid of this region. Moreover, the most stable fusion transcript (MAP4) has no “AUUUA” sequences at all, whereas the least stable fusion transcript (EIF-3) has three motifs. Thus the presented data are in agreement with the basic hypothesis that viral-cellular fusion transcripts are more stable than episome-derived transcripts [15]. However, the main cause for the difference in stability is most likely not the loss of the 3’UTR but the loss of the non-coding region between E4 and E5. The 3’UTR sequence itself does not contain “AUUUA” motifs but is AU-rich corresponding to class 3 AREs. Evidently this region sufficed to confer instability to human β-globin transcripts in cell culture [15].

Additionally to the loss of viral instability-mediating sequence elements by the integration process, the evolutionary pressure during tumorigenesis may select cells with integration loci enabling the expression of stable fusion transcripts. In-silico analyses of a set of authentic viral-cellular fusion transcript sequences from Schmitz et al. [21] revealed that 24 out of 47 fusion transcripts have no AUUUA elements (similar to MAP4) and are likely to be highly stable thereby contributing to carcinogenesis.

## 4. Materials and Methods

### 4.1. Cell Culture

To determine the stability of viral transcripts and viral-cellular fusion transcripts human foreskin keratinocytes were transduced with chimeric adenovirus particles and treated with actinomycin D to inhibit de-novo synthesis of RNA. Keratinocytes were isolated from circumcision-derived foreskins of juvenile boys [22] and cultivated under standard conditions (5% CO_2_, 37 °C, 95% humidity) in serum-free EpiLife medium supplemented with Human Keratinocyte Growth Supplement (HKGS) and 0.06 mM CaCl_2_ (Gibco, ThermoFisherScientific, Schwerte, Germany). Contaminating fibroblasts were removed by differential trypsinization, and pure keratinocyte cultures were used for all experiments. To minimize donor-specific effects on RNA stability, pools of ≥ 3 keratinocyte cultures were used for each transduction. Adenovirus constructs were generated as described below and transfected into HEK293A cells using lipofectamine (Invitrogene, ThermoFisherScientific). After 10–14 days virus particles were harvested by freeze-thaw-cycles from cells and cell culture supernatant if approximately 80% of cells showed cytopathic effects. Viral stocks were amplified in HEK293A to obtain high-titer stocks which were quantified by Adeno-X™ Rapid Titer Kit (Clontech, Saint-Germain-en-Laye, France). A multiplicity-of-infection (moi) of 80 was used for the transduction of primary keratinocytes. To this end 3.5 × 10^5^ keratinocytes were seeded in 6 cm dishes, transduced with adenoviruses and cultivated for 72 h until reaching 90% confluence. Medium was changed to actinomycin D containing medium (5 µM) and cells were harvested after different time points (0 h up to 10 h). Experiments were replicated in four independent transductions.

### 4.2. Generation of Adenovirus Constructs

Authentic viral-cellular fusion transcripts were identified in cervical cancer samples by 3’-RACE PCR (Amplification of Papillomavirus Oncogene Transcripts (APOT), [23]) and sequence analyses of aberrantly sized PCR products [16,17]. Based on this sequence information, the cDNA of three different viral-cellular fusion transcripts (Table 1) were PCR-amplified and cloned by recombinase-mediated GATEWAY-cloning into pDONR201. Verified inserts in pDONR201 clones were then subcloned into pAd/CMV/V5-DEST (Invitrogene, ThermoFisherScientific). For cDNA synthesis of episome-derived viral oncogene transcripts with and without 3’UTR (Table 1), PCR primer was derived from NCBI RefSeq:NC_001526.2. The complete sequences of all cDNAs are given in Supplementary file S1.

### 4.3. Quantification of RNA Stability

Total RNA was extracted from cells using the NucleoSpin RNA Kit (MachereyNagel, Düren, Germany) according to the manufacturer’s instructions, which include DNase treatment. The RNA concentration and quality were determined by spectrophotometry (NanoDrop ND-1000). RNAs (1 µg each) were reverse transcribed in 20 µL using oligo-dT primer (500 nM), dNTP (500 nM each), DTT (10 mM), first-strand buffer, RNaseOUT (20U) and SuperScriptIII reverse transcriptase (200U) (Invitrogen, ThermoFisherScientific). From each RNA two independent cDNA were reverse transcribed and analyzed by real-time PCR.

All real-time PCR experiments were run in duplicates on a Rotorgene cycler (Qiagen, Hilden, Germany). Reactions were performed in 25 µL volume using the FastStartUniversal SybrGreen MasterMix (Roche Diagnostics, Mannheim, Germany) containing forward and reverse primers (400 nM each) and cDNA equivalent to 50 ng RNA. Primer-specific data are listed in Table 2. The PCR steps were as follows: initial denaturation and hot-start activation at 95 °C for 10 min followed by 40 cycles of denaturation phase at 95 °C for 15 s, primer-specific annealing for 20 s at different temperatures (Table 2) and elongation at 72 °C for 40 s. Subsequently, the melting temperature of the PCR product was determined to ensure specificity. The relative amount of HPV transcripts compared to GAPDH transcript levels per time point of actinomycin D treatment was quantified by the PCR-efficiency corrected delta-C_T_ method [24]. The mean PCR efficiencies were determined for all PCR runs by the LinReg software [25]. Relative levels of HPV transcripts were normalized to time point zero and linear regression analyses revealed the time-dependent change of HPV transcripts relative to GAPDH. The slope of the regression line indicates the fold change of HPV transcripts per hour. To reduce the variation of RNA stability in independent transduction experiments by the use of keratinocytes from different donors and to enable a statistical evaluation, the relative instability of all analyzed transcripts was calculated relative to the mean of the HPV16epi-plusUTR reference in each transduction experiment.

### 4.4. HPV16-E7 Immunocytochemistry

To validate the functionality of the chimeric adenoviruses, HPV16 E7 protein expression was analyzed 72 h post-transduction. For this purpose, the cells were resuspended in PreservCyt^®^ (Cytyc Corp., Malborough, MA 01752, USA) Cytospins from 1 × 10^5^ cells were prepared by centrifugation 500 rpm, 2 min in a CellSpin centrifuge (Tharmac, Wiesbaden, Germany). Staining procedures were done according to the protocol described by Lidqvist and colleagues [26]. Briefly, the cells were permeabilized in 0.3% Triton X-100 in TBS for 15 min at room temperature. Incubation with the mouse monoclonal antibody E716-41 (Fujirebio Diagnostics, Göteborg, Schweden) at a dilution of 1 µg/mL was performed overnight at 4 °C. After several washing steps, specific staining was visualized using the Dako REAL™EnVison™ kit (Dako, Jena, Germany), which uses 3,3’-Diaminobenzidine (DAB) as a chromogen. Slides were counterstained by hematoxylin and inspected using an Axioplan microscope (Zeiss, Jena, Germany).

## Figures and Tables

**Table 1:** Characteristics of recombinant adenoviruses.

Lab Code	cDNA for Transduction	Insert Size	Additional Information
HPV16epi-plusUTR (Ref)	E6*I-E7-E1_V_E4-E5 plus 3’UTR	1477 bp	RefSeq: NC_001526.2 nt 83-4211
HPV16epi-minusUTR	E6*I-E7-E1_V_E4-E5 minus 3’UTR	1358 bp	RefSeq: NC_001526.2 nt 83-4101
HPV16-EIF1	E6*I-E7-E1_V_EIF1 exons 2_V_3_V_4 plus 3’UTR	1757 bp	Integration site 17q21.2 RefSeq: NM_005801 nt 186-1327
HPV16-MAP4	E6*I-E7-E1_V_MAP4 exons 12_v_13_v_14_v_15_v_16_v_17_v_18_v_19 plus 3’UTR	2732 bp	Integration site 3p21 RefSeq: NM_002375 nt 2602-4718
HPV16-FAM110B	E6*I-E7-E1_V_FAM110B exon 5 plus 3’UTR	3492 bp	Integration site 8q12.1 RefSeq: NM_147189 nt 588-3463

**Table 2:** Primer data.

Primer	Sequence	Product Size and Annealing Temperature
*GAPDH*-F	5′-GCGACACCCACTCCTCCACC-3′	119 bp, 58 °C
*GAPDH-*R	5′-GAGGTCCACCACCCTGTTGC-3′	
*HPV16*-F	5′-AATGTTTCAGGACCCACAGG-3′	124 bp, 58 °C
*HPV16*-R	5′-CTCACGTCGCAGTAACTGTTG-3′	

## Supplementary Materials

Supplementary materials can be found at https://www.mdpi.com/1422-0067/21/1/112/s1.

## Abbreviations

APOT	Amplification of Papillomavirus Oncogene Transcripts
ARE	Adenine and uridine rich element
CIN	Cervical epithelial neoplasia
Ct	Cycle threshold
moi	Multiplicity of infection
ncr	Non-coding region
qPCR	Quantitative PCR
RACE	Rapid amplification of cDNA ends
UTR	Untranslated region
