# Supplementary material for: Differences in Stability of Viral and Viral-Cellular Fusion Transcripts in HPV-Induced Cervical Cancers
Source: Int J Mol Sci. 2019 Dec 23;21(1):112. doi: 10.3390/ijms21010112 (PMC6981427; doi:10.3390/ijms21010112)
Supplement: Supplementary file 1 [file ijms-21-00112-s001.pdf]

Stability of viral and viral-cellular fusion transcripts in cervical cancers

Franziska Ehrig, Norman Häfner, Corina Driesch, Katrin Beer, Matthias Dürst

Department of Gynecology, Jena University Hospital – Friedrich Schiller University Jena, 07747 Jena, Germany

Supplementary File S1

Nucleotide sequences of analyzed transcripts

1. Episome-derived transcript HPV16epi-plusUTR

```
ATGCACCAAA AGAGAACTGC AATGTTTCAG GACCCACAGG AGCGACCCAG
AAAGTTACCA CAGTTATGCA CAGAGCTGCA AACAACTATA CATGATATAA
TATTAGAATG TGTGTACTGC AAGCAACAGT TACTGCGACG TGAGGTGTAT
TAACTGTCAA AAGCCACTGT GTCCTGAAGA AAAGCAAAGA CATCTGGACA
AAAAGCAAAG ATTCCATAAT ATAAGGGGTC GGTGGACCGG TCGATGTATG
TCTTGTTGCA GATCATCAAG AACACGTAGA GAAACCCAGC TGTAATCATG
CATGGAGATA CACCTACATT GCATGAATAT ATGTTAGATT TGCAACCAGA
GACAACTGAT CTCTACTGTT ATGAGCAATT AAATGACAGC TCAGAGGAGG
AGGATGAAAT AGATGGTCCA GCTGGACAAG CAGAACCGGA CAGAGCCCAT
TACAATATTG TAACCTTTTG TTGCAAGTGT GACTCTACGC TTCGGTTGTG
CGTACAAAGC ACACACGTAG ACATTCGTAC TTTGGAAGAC CTGTTAATGG
GCACACTAGG AATTGTGTGC CCCATCTGTT CTCAGAAACC ATAATCTACC
ATGGCTGATC CTGCAGAGCA ACGAAGTATC CTCTCCTGAA ATTATTAGGC
AGCACTTGGC CAACCACCCC GCCGCGACCC ATACCAAAGC CGTCGCCTTG
GGCACCGAAG AAACACAGAC GACTATCCAG CGACCAAGAT CAGAGCCAGA
CACCGGAAAC CCCTGCCACA CCACTAAGTT GTTGCACAGA GACTCAGTGG
ACAGTGCTCC AATCCTCACT GCATTTAACA GCTCACACAA AGGACGGATT
AACTGTAATA GTAACACTAC ACCCATAGTA CATTTAAAAG GTGATGCTAA
TACTTTAAAA TGTTTAAGAT ATAGATTTAA AAAGCATTGT ACATTGTATA
CTGCAGTGTC GTCTACATGG CATTGGACAG GACATAATGT AAAACATAAA
AGTGCAATTG TTACACTTAC ATATGATAGT GAATGGCAAC GTGACCAATT
TTTGTCTCAA GTTAAAATAC CAAAAACTAT TACAGTGTCT ACTGGATTTA
TGTCTATATG ACAAATCTTG ATACTGCATC CACAACATTA CTGGCGTGCT
TTTTGCTTTG CTTTGTGTGC TTTTGTGTGT CTGCCTATTA ATACGTCCGC
TGCTTTTGTC TGTGTCTACA TACACATCAT TAATAATATT GGTATTACTA
TTGTGGATAA CAGCAGCCTC TGCGTTTAGG TGTTTTATTG TATATATTAT
ATTTGTTTAT ATACCATTAT TTTTAATACA TACACATGCA CGCTTTTTAA
TTACATAATG TATATGTACA TAATGTAATT GTTACATATA ATTGTTGTAT
ACCATAACTT ACTATTTTTT CTTTTTTATT TTCATATATA ATTTTTTTTT
TTGTTTGTTT GTTTGTTTTT TAATAAA
```

Legend:

HPV-16 E6*I	nt 83-226, 409-559
HPV-16 E7	nt 562-858
HPV-16 E1	nt 865-880
HPV-16 E4	nt 3358-3620
HPV-16 E5	nt 3850-4099
Poly-A signals	AATAAA or ATTAAA
ATTTA	"AUUUA" motif, ARE (AU-rich element)
nt in black	non-coding regions e.g. UTR

2. Episome-derived transcript HPV16epi-minusUTR

```
ATGCACCAAA AGAGAACTGC AATGTTTCAG GACCCACAGG AGCGACCCAG
AAAGTTACCA CAGTTATGCA CAGAGCTGCA AACAACTATA CATGATATAA
TATTAGAATG TGTGTACTGC AAGCAACAGT TACTGCGACG TGAGGTGTAT
TAACTGTCAA AAGCCACTGT GTCCTGAAGA AAAGCAAAGA CATCTGGACA
AAAAGCAAAG ATTCCATAAT ATAAGGGGTC GGTGGACCGG TCGATGTATG
TCTTGTTGCA GATCATCAAG AACACGTAGA GAAACCCAGC TGTAATCATG
CATGGAGATA CACCTACATT GCATGAATAT ATGTTAGATT TGCAACCAGA
GACAACTGAT CTCTACTGTT ATGAGCAATT AAATGACAGC TCAGAGGAGG
AGGATGAAAT AGATGGTCCA GCTGGACAAG CAGAACCGGA CAGAGCCCAT
TACAATATTG TAACCTTTTG TTGCAAGTGT GACTCTACGC TTCGGTTGTG
CGTACAAAGC ACACACGTAG ACATTCGTAC TTTGGAAGAC CTGTTAATGG
GCACACTAGG AATTGTGTGC CCCATCTGTT CTCAGAAACC ATAATCTACC
ATGGCTGATC CTGCAGAGCA ACGAAGTATC CTCTCCTGAA ATTATTAGGC
AGCACTTGGC CAACCACCCC GCCGCGACCC ATACCAAAGC CGTCGCCTTG
GGCACCGAAG AAACACAGAC GACTATCCAG CGACCAAGAT CAGAGCCAGA
CACCGGAAAC CCCTGCCACA CCACTAAGTT GTTGCACAGA GACTCAGTGG
ACAGTGCTCC AATCCTCACT GCATTTAACA GCTCACACAA AGGACGGATT
AACTGTAATA GTAACACTAC ACCCATAGTA CATTTAAAAG GTGATGCTAA
TACTTTAAAA TGTTTAAGAT ATAGATTTAA AAAGCATTGT ACATTGTATA
CTGCAGTGTC GTCTACATGG CATTGGACAG GACATAATGT AAAACATAAA
AGTGCAATTG TTACACTTAC ATATGATAGT GAATGGCAAC GTGACCAATT
TTTGTCTCAA GTTAAAATAC CAAAAACTAT TACAGTGTCT ACTGGATTTA
TGTCTATATG ACAAATCTTG ATACTGCATC CACAACATTA CTGGCGTGCT
TTTTGCTTTG CTTTGTGTGC TTTTGTGTGT CTGCCTATTA ATACGTCCGC
TGCTTTTGTC TGTGTCTACA TACACATCAT TAATAATATT GGTATTACTA
TTGTGGATAA CAGCAGCCTC TGCGTTTAGG TGTTTTATTG TATATATTAT
ATTTGTTTAT ATACCATTAT TTTTAATACA TACACATGCA CGCTTTTTAA
TTACATAA
```

```
Legend:
HPV-16 E6*I       nt 83-226, 409-559
HPV-16 E7         nt 562-858
HPV-16 E1         nt 865-880
HPV-16 E4         nt 3358-3620
HPV-16 E5         nt 3850-4099
Poly-A signals    AATAAA or ATTAAA
ATTTA             "AUUUA" motif, ARE (AU-rich element)
nt in black       non-coding regions e.g. UTR
```

3. Fusion transcript HPV16-MAP4

```
ATGCACCAAA AGAGAACTGC AATGTTTCAG GACCCACAGG AGCGACCCAG
AAAGTTACCA CAGTTATGCA CAGAGCTGCA AACAACTATA CATGATATAA
TATTAGAATG TGTGTACTGC AAGCAACAGT TACTGCGACG TGAGGTGTAT
TAACTGTCAA AAGCCACTGT GTCCTGAAGA AAAGCAAAGA CATCTGGACA
AAAAGCAAAG ATTCCATAAT ATAAGGGGTC GGTGGACCGG TCGATGTATG
TCTTGTTGCA GATCATCAAG AACACGTAGA GAAACCCAGC TGTAATCATG
CATGGAGATA CACCTACATT GCATGAATAT ATGTTAGATT TGCAACCAGA
GACAACTGAT CTCTACTGTT ATGAGCAATT AAATGACAGC TCAGAGGAGG
AGGATGAAAT AGATGGTCCA GCTGGACAAG CAGAACCGGA CAGAGCCCAT
TACAATATTG TAACCTTTTG TTGCAAGTGT GACTCTACGC TTCGGTTGTG
CGTACAAAGC ACACACGTAG ACATTCGTAC TTTGGAAGAC CTGTTAATGG
GCACACTAGG AATTGTGTGC CCCATCTGTT CTCAGAAACC ATAATCTACC
ATGGCTGATC CTGCAGCCAT TAAGACTGAG GGAAAACCTG CAGAAGTCAA
GAAGATGACT GCAAAGTCTG TACCAGCTGA CTTGAGTCGC CCAAAGAGCA
CCTCCACCAG TTCCATGAAG AAAACCACCA CTCTCAGTGG GACAGCCCCC
GCTGCAGGGG TGGTTCCCAG CCGAGTCAAG GCCACACCCA TGCCCTCCCG
GCCCTCCACA ACTCCTTTCA TAGACAAGAA GCCCACCTCG GCCAAACCCA
GCTCCACCAC CCCCCGGCTC AGCCGCCTGG CCACCAATAC TTCTGCTCCT
GATCTGAAGA ATGTCCGCTC CAAGGTTGGC TCCACGGAAA ACATCAAGCA
TCAGCCTGGA GGAGGCCGGG CCAAAGTAGA GAAAAAAACA GAGGCAGCTG
CTACAACCCG AAAGCCTGAA TCTAATGCAG TCACTAAAAC AGCCGGCCCA
ATTGCAAGTG CACAGAAACA ACCTGCGGGG AAAGTCCAGA TAGTCTCCAA
AAAAGTGAGC TACAGCCATA TTCAGTCCAA GTGTGGTTCC AAGGACAATA
TTAAGCATGT CCCTGGAGGT GGTAATGTTC AGATTCAGAA CAAGAAAGTG
GACATCTCTA AGGTCTCCTC CAAGTGTGGG TCTAAGGCTA ACATCAAGCA
CAAGCCTGGT GGAGGAGATG TCAAGATTGA AAGTCAGAAG TTGAACTTCA
AGGAGAAGGC CCAGGCCAAG GTGGGATCCC TCGATAATGT GGGCCACCTA
CCTGCAGGAG GTGCTGTGAA GACTGAGGGC GGTGGCAGCG AGGCTCCTCT
GTGTCCGGGT CCCCCTGCTG GGGAGGAGCC GGCCATCTCT GAGGCAGCGC
CTGAAGCTGG CGCCCCCACT TCAGCCAGTG GCCTCAATGG CCACCCCACC
CTGTCAGGGG GTGGTGACCA AAGGGAGGCC CAGACCTTGG ACAGCCAGAT
CAGGAGACAA GCATCTAATG ATGACATTCT GGTCTCGTCT TCCGTCTCCC
CCGTGTTCCC CTCTTGTCTC CCCTGTTCCC CTCTCCCTTC CCTCCTCCCA
TGTCACTGCA GATTGAGACC TACAGGCTGA CGTTCCGGGC AAATGCCAGG
GCCCGCACCG ACCACGGGGC CGACATTGTC TCCCGCCCCC CACACTTCCC
TGGCGGCCCC AACTCGGGCT CCCGGGTCCT TGGCCCCCTT TCCCGGGCTG
TCCACTAGAC CAGTGAGCGC TTGGGCGCCG TGCTGGGCAG CCCGCTAGGC
TCGCCTTCCC TCCTGCTTTG CGTGCCCGGG GCAGCAGCAG CCCTGCCCCA
CACCTCCTCT CACTCCCCAG CCTGGGCCCA TCTCCCTGCT TTGGTCTTGC
CCCATCACTG CGCCACTGCT CCGTGGAGGA GGTTGGGAGG GGGTTGGGGT
GGTTGAGGCT AAGTTGGGAT CTAGGAGAGG AGAACCAGAT TCTATCCTCA
TCTTTTTTTG GTTCTTTGGT CCAAACCCAA AAGAAACTGA CATGCCCTCC
CTTCTCCCTG GATCTACCTG GAGGGAAGAG TGGAGGTGGA TTCCGAGTGG
TGACAGGACG CTGACCGTGG AGCTTAAGCC ACTGCCTCTC CCTCTGGTCC
CACAAATGGG CGCCCCCCCC TCCCCATGCA GGTGGTGTCG GGCCCTTCTT
GCTGCCCTGC CCCAAGTTGG GGGTCAGTGC TGCCTGTCCC CATGCTTAAC
ATACCCGCCT AGCTGCTGTC ACATTTTTCT TGTTTTGTCC TTTTATTTTT
TTCTAATAAC CTAAAAACTG GCAAAATAGT TCTGCAGGTT GAAGCCATGT
CTACATGAAA GTCCTCAGTA AGTGTTAGAG GGAACAGGGC GGAGATATCC
TTATGCCACC CCCGCTGGAG GATGTGGGCA GCTTAGGGCC CTGGAGGCGG
TGCGGCAGGG AAGAGGGGTG CAGAGGCTGT GGCTGGTGAG CCGGTCAGGC
ACACAAGGGG CCCTTGGAGC GTGGACTGGT TGGTTTTGCC ATTTTGTTGT
GTGTATGCTG CTTTTCTTTT CTAACCAAGA GGCTGGTTTT GGCATCTCTG
TCCCATTCCC TGGGATCTGG TGGTCAGCCC TAGGATAAAA AGCCAGGGCT
GGAGAACAAG AAAGGGCCAG GAGATGGAAT TC
```

Legend:

HPV-16 E6*I	nt 83-226, 409-559
HPV-16 E7	nt 562-858
HPV-16 E1	nt 865-880
cellular **exons**	
Poly-A signals	AATAAA or ATTAAA
ATTTA	"AUUUA" motif, ARE (AU-rich element)
nt in black	non-coding regions e.g. UTR

4. Fusion transcript HPV16-EIF1

```
ATGCACCAAA AGAGAACTGC AATGTTTCAG GACCCACAGG AGCGACCCAG
AAAGTTACCA CAGTTATGCA CAGAGCTGCA AACAACTATA CATGATATAA
TATTAGAATG TGTGTACTGC AAGCAACAGT TACTGCGACG TGAGGTGTAT
TAACTGTCAA AAGCCACTGT GTCCTGAAGA AAAGCAAAGA CATCTGGACA
AAAAGCAAAG ATTCCATAAT ATAAGGGGTC GGTGGACCGG TCGATGTATG
TCTTGTTGCA GATCATCAAG AACACGTAGA GAAACCCAGC TGTAATCATG
CATGGAGATA CACCTACATT GCATGAATAT ATGTTAGATT TGCAACCAGA
GACAACTGAT CTCTACTGTT ATGAGCAATT AAATGACAGC TCAGAGGAGG
AGGATGAAAT AGATGGTCCA GCTGGACAAG CAGAACCGGA CAGAGCCCAT
TACAATATTG TAACCTTTTG TTGCAAGTGT GACTCTACGC TTCGGTTGTG
CGTACAAAGC ACACACGTAG ACATTCGTAC TTTGGAAGAC CTGTTAATGG
GCACACTAGG AATTGTGTGC CCCATCTGTT CTCAGAAACC ATAATCTACC
ATGGCTGATC CTGCAGACCC CTTTGCTGAT GCAAGTAAGG GTGATGACCT
GCTTCCTGCT GGCACTGAGG ATTATATCCA TATAAGAATT CAACAGAGAA
ACGGCAGGAA GACCCTTACT ACTGTCCAAG GGATCGCTGA TGATTACGAT
AAAAAGAAAC TAGTGAAGGC GTTTAAGAAA AAGTTTGCCT GCAATGGTAC
TGTAATTGAG CATCCGGAAT ATGGAGAAGT AATTCAGCTA CAGGGTGACC
AACGCAAGAA CATATGCCAG TTCCTCGTAG AGATTGGACT GGCTAAGGAC
GATCAGCTGA AGGTTCATGG GTTTTAAGTG CTTGTGGCTC ACTGAAGCTT
AAGTGAGGAT TTCCTTGCAA TGAGTAGAAT TTCCCTTCTC TCCCTTGTCA
CAGGTTTAAA AACCTCACAG CTTGTATAAT GTAACCATTT GGGGTCCGCT
TTTAACTTGG ACTAGTGTAA CTCCTTCATG CAATAAACTG AAAAGAGCCA
TGCTGTCTAG TCTTGAAGTC CCTCATTTAA ACAGAGGTCA AGCAATAGGC
GCCTGGCAGT GTCAAGCCTG AAACCAAGCA ATACCGTCAT GTTTCAGCCA
AGCCCAGAGC CCTAAGATTA CAAACAACTA TGGCCGGAAC CTCCTCAGCT
CTCCCTCTGC AGAGTTCCCT ACCCTAAGAG AATGTTACCA CCTGAACAGT
CCTCGGTGAA TCTGAGAGGA GAGGATGGGG TAAGGCAGAA GCACCAGCTG
TACTACTAGA AGGAGCTTTT GGTGGTAGAT CCCCTGGTGT CTCCAACCTG
ACTAGGTGGA CAGAGCTCAA AGAGGCCCTC TTACCGCTAG CGAGGTGATA
GGACATCTGG CTTGCCACAA AGGTCTGTTC GACCAGACAT ATCCTAGCTA
AGGGATGTCC AAACATCAGA ATGTGAGGCC AACCTTCTAT CAGAGTTAAA
CTTTTGACAA GGGAACAAAT CTCAAACTGA TCCATCAGTC ATGTAGCTAG
CTGTAGAGCT TGCAACTTAA TAGCAGCAGC TGCCCAATGC CATGTGAAGT
AACAAACTGG TTTTTGGTTT TTTTTTCCCC TTCAGTTTTA ATGTTATGTG
TAATGTATTT AAACCCTTAT TTAAATAAAA CTTGTTTTCA GAAATACCTG
ACTTGCA
```

Legend:

HPV-16 E6*I	nt 83-226, 409-559
HPV-16 E7	nt 562-858
HPV-16 E1	nt 865-880
cellular **exons**	
Poly-A signals	AATAAA or ATTAAA
ATTTA	"AUUUA" motif, ARE (AU-rich element)
nt in black	non-coding regions e.g. UTR

5. Fusion transcript HPV16-FAM110B

ATGCACCAAA AGAGAACTGC AATGTTTCAG GACCCACAGG AGCGACCCAG
AAAGTTACCA CAGTTATGCA CAGAGCTGCA AACAACTATA CATGATATAA
TATTAGAATG TGTGTACTGC AAGCAACAGT TACTGCGACG TGAGGTGTAT
TAACTGTCAA AAGCCACTGT GTCCTGAAGA AAAGCAAAGA CATCTGGACA
AAAAGCAAAG ATTCCATAAT ATAAGGGGTC GGTGGACCGG TCGATGTATG
TCTTGTTGCA GATCATCAAG AACACGTAGA GAAACCCAGC TGTAATCATG
CATGGAGATA CACCTACATT GCATGAATAT ATGTTAGATT TGCAACCAGA
GACAACTGAT CTCTACTGTT ATGAGCAATT AAATGACAGC TCAGAGGAGG
AGGATGAAAT AGATGGTCCA GCTGGACAAG CAGAACCGGA CAGAGCCCAT
TACAATATTG TAACCTTTTG TTGCAAGTGT GACTCTACGC TTCGGTTGTG
CGTACAAAGC ACACACGTAG ACATTCGTAC TTTGGAAGAC CTGTTAATGG
GCACACTAGG AATTGTGTGC CCCATCTGTT CTCAGAAACC ATAATCTACC
ATGGCTGATC CTGCAGCTCT GCCTGCAGTT GAACACAAAG ACCTGGAGGA
GACTCCCACCT CCTTCAGGGA GAAGAAAGGA GGCAGCGAAG CAGATTAAAG
GAACTTGTGG CTTGTGGCCT TTTTGTGCAA GGGCCGCCTG GAAGGGGAGA
AAAGTGTATG AAAGCCACCG TGGCGGTTAA TGAGCTGTGA GATGAGGCGC
TGCTGCGGCC GCTGCTGCTG ACACTCGCTC CCAGGCTGCA CCCGCCGCCC
TTGGCGCTTC ATGTACATGT GTCTATTCAG GCCTTGCGGA GGCGCCCAGA
AGAGCATCCA ACACGGCTGC CGGGGAAAGA CCGCCCACCA TGCCCACGGA
GACCCTACAG ACAGGTAGCA TGGTGAAGCC GGTCAGCCCC GCGGGCACCT
TCACCTCTGC TGTGCCCCTG CGCATCCTGA ACAAGGGGCC AGACTACTTC
CGCAGGCAGG CCGAGCCCAA CCCCAAGAGG CTCAGCGCCG TGGAGAGGCT
GGAGGCCGAC AAGGCCAAGT ACGTCAAGAG CCAGGAGGTG ATCAACGCCAA
GCAGGAGCCC GTGAAGCCCG CCGTGCTGGC CAAGCCCCCG GTGTGCCCGG
CTGCCAAGCG CGCACTGGGC AGCCCCACGC TCAAAGTGTT CGGCAACCAC
GCCAAGACCG AGAGCGGCGT GCAGAGGGAG AACCTGAAGC TGGAGATCCT
GAAGAACATC ATCAATAGCT CCGAGGGCTC TAGCTCGGGC TCGGGGCACA
AGCACAGCTC CCGCAACTGG CCGCCCCACC GGTCGGAAGC CACTGACCTG
CACCGTCACT CCTTCGCGGA GTCCCTGAAG GTCTACCCCA CGCAGGGCCG
CAGGAGCCCG CAGGAGGGCG GCTCCCACGT GGGCAGGAGA CTGCTGGAGC
AGTCAGCCGA GTCCTTCCTC CACGTGTCCC ACAGCTCTTC GGACATCCGC
AAGGTGACCA GCGTGAAGCC CCTCAAGGCC ATCCCCTGCA GTAGCTCTGC
CCCTCCCCTG CCTCCCAAGC CCAAAATCGC AGCCATCGCC TCCATGAAGT
CCCCCGAGGC CGACCCTGTG GAACCAGCTT GTGGAGTCAG CCGAAGACCC
TCCCTCCAGC GGTCTAAGTC AGACTTGAGT GACAGATATT TCCGAGTGGA
CGCGGACGTG GAGAGGTTC TTCAACTACT GTGGACTGGA CCCGGAAGAG
CTGGAAAACC TGGGAATGGA AAACTTTGCA AGGGCTAATT CTGACATAAT
ATCCCTCAAC TTCCGCAGCG CAAGCATGAT CAGCTCAGAC TGTGAACAGT
CTCAGGACAG TAACAGTGAC CTTAGAAATG ATGACAGTGC CAATGACCGC
GTGCCGTATG GCATTTCTGC CATCGAAAGA AATGCTAGAA TCATCAAGTG
GTTATATAGC ATCAAACAAG CTAGAGAGTC ACAGAAGGTC TCCCATGTGT
AAGACAGTGC GTGGAAAGGA GGGAGCGTGG GTCTCTGTGC TTACGCAGTT
GTGAAGGTTG TGAGGTCTCC TTGAAGTGTT GTGAGCTTCT CCACTCTTTG
TGTTGCTTGT TGTGCAATGT TTTCAAGTTG CATGCTTGTC AACATGGGGA
CTGACCTGGC TTCCACGTCA CCATCGCTAC AGAGCCTGGC TGAACACGCG
TCATCTGCCA AAAGTTTCAG GCCAGAATCT AATTAGGGCT TTGCTCGTAA
GCAAAACTGT TTACATGAAA ATGGTATACA ACTTCTTCGA TATTTATGTG
GTTCTTGTGT TTTTATATCC CGCGCTATTG TGTCTTAATT AAAAGTTTTA
TCAACTGGCT TTTAAGTTGA GAGCAGAATC TTTTTGAAAT AAAAAGCCAC
TTTGCCTACA TGTGAGACTA TTCCAGTGGG ACAGTAAATG GGATCTGCTA
CCAAACAGAT AATGACTGAC CGAACATAGA ACCAGGCTGG GTTTTCTGTG
GAATAAAGTG GTGAGCCTGA CGTTAATCGT TTACATGTTG AAATTCTCTT
GCCACTTAGT GAAGCAGTCA TTGGGGTAAC AATGCTACGT TTTGTGTCCG
ATTCAGTGTG TAAACGTTGG TGGTTCTATG TAATACTGAC CCCAAGGAAA
GACGATCAGA TACAAAGAAA GATGGTTAAA TCTAGGAGGT TGGGTGTTTG
GGGGTTTTGT TGATGTTGTT CTGATTGGGA TAATTCAGAA ATTCCTAATA
TTATCTTTCG ACAGAATGAC CACCCTAAAA CAAATACTGA ATGCCTTTAA
AAAAAAAAAA AGTTGTGGTT TTTTGTTTTT TTTTTTTTTT TTTTTGGTCG
AGAACTACTA ATTTCATGCT ATTTCTTTCT CTCTCCCTTT GTTGTTATTT
GATTTGGGGG AAGGGAGTGA GGCTTGTGCA GATTTGAGCT ATCTGAAGAC
ATGAACAGAA GTCAAATACC AGCCATATCA GTAGAGCCTT CCATTCAAAA
GTGAACACAC TTGGTAGCTG AACCATATCT GAGCAGCGTG ATTCTGTTGT

```
CTTGCATATG TTATTCTCTC AGGCTGTGGA TCTTTGTTAC AGCTCTAGGA
AACTGTAGAA ATACAATGCT ATGTAGCTTT TCCACCCCAT GGTCTCTAGT
GCTGCCTATC AACTTGTCCC CTTTTTTCCC AATAACCTGT CTTCCAGGTG
GTACAGTTAG CTGTCACTCA GCTGACACCA TGATGTGGCA GCAGAGAGGG
AAACCTACAA GTGGTTTGCC TCATTGCCTT TGCCACATCT GAAGTTCTCA
GCAGCACTAC CTTAGACTTC ATGAGCTAAT AGGAAACTTT TTATGGTGTA
AATGCTGTAA GACTTTGTAC ATACTTCAGT TGTTATGAAA TCTTTAAAGA
AAAAAAGAAA AAGTTACGCT AATAAATTGC TGTGGTGCAG G
```

Legend:

HPV-16 E6*I	nt 83-226, 409-559
HPV-16 E7	nt 562-858
HPV-16 E1	nt 865-880
cellular **exons**	
Poly-A signals	AATAAA or ATTAAA
ATTTA	"AUUUA" motif, ARE (AU-rich element)
nt in black	non-coding regions e.g. UTR
